# A potential role of heat‐moisture couplings in the range expansion of *Striga asiatica*

**DOI:** 10.1002/ece3.11332

**Published:** 2024-05-16

**Authors:** Marco Bürger, Joanne Chory

**Affiliations:** ^1^ Plant Biology Laboratory Salk Institute for Biological Studies La Jolla California USA; ^2^ Howard Hughes Medical Institute, Salk Institute for Biological Studies La Jolla California USA

**Keywords:** agricultural productivity, climate change, *Orobanche*, parasitic weeds, *Striga*

## Abstract

Parasitic weeds in the genera *Orobanche*, *Phelipanche* (broomrapes) and *Striga* (witchweeds) have a devastating impact on food security across much of Africa, Asia and the Mediterranean Basin. Yet, how climatic factors might affect the range expansion of these weeds in the context of global environmental change remains unexplored. We examined satellite‐based environmental variables such as surface temperature, root zone soil moisture, and elevation, in relation to parasitic weed distribution and environmental conditions over time, in combination with observational data from the Global Biodiversity Information Facility (GBIF). Our analysis reveals contrasting environmental and altitude preferences in the genera *Striga* and *Orobanche*. Asiatic witchweed (*Striga asiatica*), which infests corn, rice, sorghum, and sugar cane crops, appears to be expanding its range in high elevation habitats. It also shows a significant association with heat‐moisture coupling events, the frequency of which is rising in such environments. These results point to geographical shifts in distribution and abundance in parasitic weeds due to climate change.

## Graphical abstract

Our research examines the effects of climate change on the distribution of parasitic weeds, such as *Orobanche*, *Phelipanche*, and *Striga*, which pose threats to global food security. Utilizing satellite data and GBIF observations, we find climate‐induced shifts in habitats and expansion patterns, notably the elevation increase of *Striga asiatica*, emphasizing the growing impact of climate variables on weed distribution.

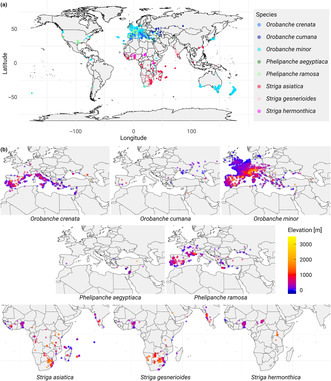

## 1 INTRODUCTION

Parasitic weeds in the genera *Orobanche*, *Phelipanche* (broomrapes) and *Striga* (witchweeds) pose a substantial challenge to global agricultural productivity (Joel et al., 2007; Parker, 2012; Rubiales, 2020). Each year, they devastate approximately 50–100 million hectares of farmland worldwide, causing economic losses amounting to at least $7 billion in Africa alone and impacting the livelihoods of over 300 million people (Ejeta & Gressel, 2007; Spallek et al., 2013), especially in Sub‐Saharan Africa. More than half of the cereal‐producing arable land in Sub‐Saharan Africa suffers from infestations by Striga (Gressel et al., 2004; Rodenburg et al., 2016). These parasitic plants have particularly severe effects on staple crops such as rice, maize (Nyakurwa et al., 2018), sorghum (Gurney et al., 2002), finger millet (Midega et al., 2010), and pearl millet (Wilson et al., 2004). This parasitism leads to significant yield reductions, exacerbating food insecurity issues (Makaza et al., 2023; Rodenburg et al., 2016). *Striga hermonthica*, commonly known as purple witchweed, thrives in tropical savannah conditions, characterized by warm temperatures and rainfall. *Striga asiatica*, or red witchweed, is indigenous to Asia and Africa and typically favors subtropical to tropical climates, often inhabiting sandy or loamy soils. *Striga gesnerioides*, known as cowpea witchweed, shares a similar geographical distribution with *S. asiatica* but shows unique adaptations to different hosts, environments, and agricultural systems across its range (Joel et al., 2013). On the other hand, *Orobanche* species primarily inhabit the sub‐tropical and mildly temperate regions of the Northern Hemisphere. *Orobanche crenata*, or bean broomrape, *Phelipanche ramosa* (branched broomrape), and *Phelipanche aegyptiaca* (Egyptian broomrape) are devastating parasitic weeds in the Mediterranean and Middle East regions (Grenz & Sauerborn, 2007; Mohamed et al., 2006), posing a substantial threat to fava beans (Fernandez‐Aparicio et al., 2016; Pérez‐de‐Luque et al., 2010), peas (Arjona‐Berral et al., 2006), lentils (En‐nahli et al., 2021; Rubiales et al., 2008), and other legume crops as well as crops in the Solanaceae, Brassicaceae, and Apiaceae. *Orobanche crenata* has been extending its range into east African countries (e.g. Ethiopia and Sudan (Parker, 2012)), as well as into European regions that more distant from the Mediterranean, with reports of its presence in Central Spain (Rubiales et al., 2008) and England (Parker, 2014). *Orobanche cumana*, or sunflower broomrape, endangers sunflower crops in all major sunflower‐producing countries from Europe to Asia (Molinero‐Ruiz et al., 2015).

The life cycles of these plants include a preconditioning phase (Brown & Edwards, 1946) that allows their seeds to respond to host‐exuded strigolactones and some other small‐molecule germination stimulants (Nelson, 2021). *Orobanche* and *Striga* seeds can lie dormant in the soil for decades until suitable environmental conditions induce seed preconditioning (Casadesus & Munne‐Bosch, 2021; Joel et al., 2013; Kebreab & Murdoch, 1999). However, suboptimal preconditioning parameters may substantially decrease seed germination rates or even prevent germination altogether. Distinct species within these genera exhibit different preconditioning preferences: *Orobanche* species tend to favor moderate temperature conditions (between 10°C and 20°C), while *Striga* species have an optimal preconditioning response at higher temperature (between 25°C and 35°C) and moisture levels (Aflakpui et al., 1998; Babiker et al., 2001; Eizenberg et al., 2001; Matusova et al., 2004). These differing preferences suggest that shifting climate patterns could also impact these species. Models using climate data have mainly focused on predicting future habitats and have indeed projected worrying scenarios regarding these parasites' potential spread: *Striga* species are expected to significantly expand their range in Africa if current climate trends continue (David et al., 2022), and multiple *Orobanchaceae* species could threaten all tropical and subtropical regions globally (Mohamed et al., 2006). However, how these species' preconditioning phases and germination dynamics respond to variable weather patterns and shifting climate trends remains an open question.

**FIGURE 1:**
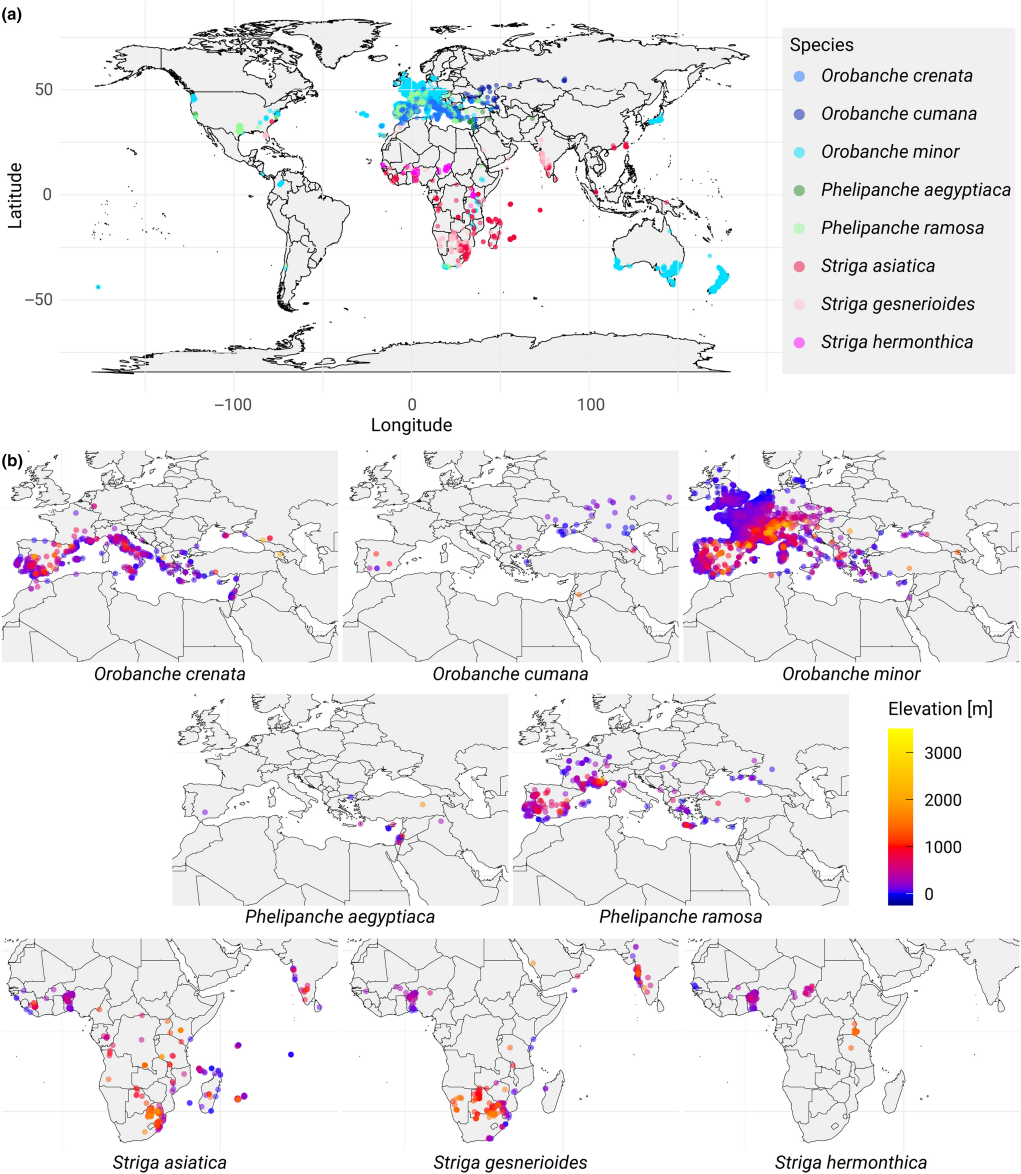
Distribution of selected Orobanchaceae species from 2004 to 2022. (a) Global observations of different Orobanchaceae species. (b) Regional observations of these species colored by elevation.

Various climatic variables influence soil conditions, land surface temperature (LST) and root‐zone soil moisture (RZSM), are particularly pivotal due to their direct impact on germination processes, and are available in time‐resolved, global datasets. In contrast, soil temperature, often requiring in situ measurements, lacks the global coverage achievable with satellite‐based LST data. Solar radiation, crucial for photosynthesis in most plants, is less informative for parasitic plants with limited photosynthetic activity. While global precipitation data indicate water input, they do not account for variables like evaporation or runoff, making RZSM a more direct measure of soil moisture availability for plant uptake. Other parameters such as wind speed and direction, though available, present considerable challenges in correlating with the timing of seed availability for dispersal and germination. In addition, elevation plays a crucial role in determining the ecological niches and distribution patterns of plant species, including those within the Orobanchaceae family. As elevation increases, climatic conditions, such as temperature and moisture availability, can change, impacting the growth of both the parasitic weeds and their host species. Weedy Orobanchaceae cover a wide range of elevation over large geographical areas (Figure 1). Elevation might influence plant interactions, germination rates, and overall prevalence; therefore, understanding the influence of elevation could be essential for predicting changes in the distribution of parasitic weeds in response to climate change and for developing effective management strategies.

Here, we aim to achieve a new perspective on the impact of environmental changes on agriculturally relevant parasitic Orobanchaceae and their host plants worldwide. To this end, we compiled and analyzed every available global observation data point—totaling approximately 13,000— of *Orobanche crenata*, *O. cumana*, *O. minor*, *Phelipanche aegyptiaca*, *P. ramosa*, *Striga asiatica*, *S. gesnerioides*, and *S. hermonthica* covering the last 19 years. We correlated these observations with environmental factors that collectively influence germination, growth, and survival such as land surface temperature, root‐zone soil moisture, and elevation. The insights gained from this analysis are intended to inform more effective management strategies for controlling these species in agricultural settings.

## 2 METHODS

### 2.1 Data collection and processing

Data on the occurrences of the *Orobanche*, *Phelipanche*, and *Striga* species were obtained from the Global Biodiversity Information Facility (GBIF). The following datasets were created:

For *Orobanche* species: GBIF.org (02 May 2023) GBIF Occurrence Download https://doi.org/10.15468/dl.hdjf3h.

For *Phelipanche* species: GBIF.org (02 May 2023) GBIF Occurrence Download https://doi.org/10.15468/dl.sn54fx.

For *Striga* species: GBIF.org (02 May 2023) GBIF Occurrence Download https://doi.org/10.15468/dl.2hdkfj.

Duplicate data entries and entries with missing or uncertain geocoordinates or species identifications were purged. Data entries that were incomplete for the different analyses, such as lacking LST data for the temperature analysis, missing RZSM data for the root‐zone soil moisture analysis, or missing either for the heat‐moisture coupling analysis, were excluded.

### 2.2 General data analysis

The R software environment version 4.1.1 (R.‐Core‐Team, 2021) was used for all data analysis and visualization. All used packages were R packages.

### 2.3 Land surface temperature (LST) retrieval

Terra Moderate Resolution Imaging Spectroradiometer (MODIS) Land Surface Temperature and Emissivity (LST&E) version 6 data from 2001 to 2022 was used as a monthly average LST dataset at a spatial resolution of 0.05 degrees, which is approximately a 5.6 × 5.6 km grid at the equator. Geographic data format packages ‘rgdal’ (Bivand et al., 2022) and ‘gdalUtils’ were used to read the raw HDF files. The raw data were then converted into a raster format using the ‘raster’ and ‘terra’ packages. After converting the temperature units from Kelvin to Celsius, the ‘raster’ package was used to extract temperature values for specific geographic coordinates corresponding to the plant observation coordinates. Data transformation and visualization were facilitated by the ‘dplyr’ and ‘ggplot2’ packages, while ‘ggmap’, ‘rasterVis’, ‘RColorBrewer’, and ‘gplots’ were employed for data exploration.

### 2.4 Root‐zone soil moisture (RZSM) retrieval

Gravity Recovery and Climate Experiment (GRACE) satellites indirectly infer root‐zone soil moisture (RZSM) by measuring changes in Earth's gravity field, which relate to variations in terrestrial water storage. The spatial resolution of GRACE data, after post‐processing, is approximately 100,000 km^2^. GRACE provides monthly data and has been successfully used to, for example, measure rates of groundwater depletion in California's Central Valley (Famiglietti et al., 2011). We used GRACE data for root‐zone soil moisture (RZSM) spanning from 2001 to 2022. The data were obtained from the NASA GRACE Global Data Archive, which is hosted by the National Drought Mitigation Center at the University of Nebraska‐Lincoln. The ‘ncdf4’ package (Pierce, 2022) was used to read the .nc4 file format of the GRACE data. Data transformation was carried out, including rasterizing the raw data with the ‘raster’ package, and flipping the rasters for correct geospatial orientation. RZSM values were then extracted for specific coordinates corresponding to the plant observations.

### 2.5 Elevation data retrieval

The ‘rgbif’ package (Chamberlain et al., 2023) was utilized to access the elevation information of each recorded plant observation coordinate. This process involved the usage of the ‘elevation’ function, which extracts elevation data from the Shuttle Radar Topography Mission's (SRTM) 3 arc‐second global digital elevation model, which provides elevation information at a resolution of approximately 90 m. The SRTM was a specially modified radar system that flew onboard the Space Shuttle Endeavour, providing interferometric C‐band synthetic aperture radar data that was used to produce a map of the Earth's land surface topology. SRTM data used in this study are publicly available through several R packages that use a HTTP protocol to request data from the server where the SRTM data is hosted, or it can be accessed through the NASA Earthdata Search.

### 2.6 Analysis and visualization of environmental variables

For the comparison of LST, RZSM, and elevation among Orobanchaceae species, we utilized the ‘dplyr’ package for data filtering and summarizing, and ggplot2 for visualization. An ANOVA, performed with base R functions, assesses significant differences across species. This was followed by Tukey's HSD test for identifying specific differences. The results are visualized through box plots with added jitter points for clarity, created using ‘ggplot2’, and the ‘multcompView’ package was used to annotate the plots with significant difference letters from Tukey's test. In order to analyze and visualize the variation within the Orobanchaceae species dataset through principal component analysis (PCA), we used the ‘tidyverse’ package for data manipulation and filtering, allowing the selection of specific columns and handling of missing values. The ggplot2 package was then utilized in conjunction with ‘factoextra’. We performed PCA on multiple subsets of the data, each representing different species, using the ‘prcomp’ function.

### 2.7 Analysis of elevation trends for *Striga* and *Orobanche* species

Observational data for *Striga* and *Orobanche* species were examined to investigate changes in elevation these species occupy over time. For each observation, a binary variable was created indicating whether the recorded elevation exceeded 1000 m. An elevation of 1000 m above sea level is a commonly used benchmark in ecological and biogeographical studies to differentiate between lowland and montane environments (Brooks et al., 2001; Huang et al., 2012; Shearman & Bryan, 2011). A logistic regression model was then fit to these data, with the year of observation serving as the predictor variable and the high‐elevation binary variable as the response. This analysis allowed for the quantification of the temporal trend in the likelihood of these species being observed above 1000 m. The estimated regression coefficients were converted into odds ratios and probabilities to provide additional insight into the changes over time.

### 2.8 Extraction and integration of geo‐spatial crop data

For plant geocoordinates, crop data were derived from geotiff files from Earthstat (year 2000) and GAEZ+ (year 2015) datasets, which contained information about harvested areas for different crops. Earthstat is a collaboration between the Global Landscapes Initiative at the University of Minnesota's Institute on the Environment and the Land Use and Global Environment Lab at the University of British Columbia. The Earthstat dataset for the year 2000 was chosen because it provides a global data set of croplands and pastures circa 2000 by combining agricultural inventory data and satellite‐derived land cover data. GAEZ+ is an update to the Global Agro‐Ecological Zones (GAEZ) Version 4 data of circa 2010 crop harvested area. It provides global, gridded (5‐arcminute resolution) irrigated and rainfed crop harvested areas, irrigated and rainfed crop production, and irrigated and rainfed crop yield for 26 different crops and crop categories. The GAEZ+ dataset for the year 2015 was chosen because it provides more recent data on production, yield, and harvested areas.

### 2.9 Analysis of heat‐moisture coupling preceding *Striga* and *Orobanche* observations

The occurrence dataset was processed to generate new binary variables indicating whether a heat‐moisture coupling event occurred in the month of each observation or in either of the two preceding months. We defined a heat‐moisture coupling event as an occurrence where the LST is ≥25°C and the RZSM percentile is ≥70. These cutoffs were chosen to ensure a temperature that allows for successful seed pre‐conditioning and germination (Aflakpui et al., 1998; Babiker et al., 2001; Matusova et al., 2004), as well as a soil‐moisture level in the approximate upper tercile, which is generally considered wet soil (Li et al., 2022; Tian & Zhang, 2023). Any instances with missing values were excluded during the cleaning process. A final binary variable was then produced, indicating whether a heat‐moisture coupling event occurred at any of the specified time points. The dataset was subsequently grouped by species, and the percentages of observations with and without a preceding heat‐moisture coupling were calculated.

### 2.10 Temporal trend analysis of land surface temperature and root zone soil moisture

Monthly LST and RZSM values were compiled for selected high‐altitude locations. To investigate monotonic trends over the study period, a non‐parametric Mann‐Kendall test was utilized.

## 3 RESULTS

### 3.1 Parasitic weed habitats vary in surface temperature and soil moisture

**FIGURE 2:**
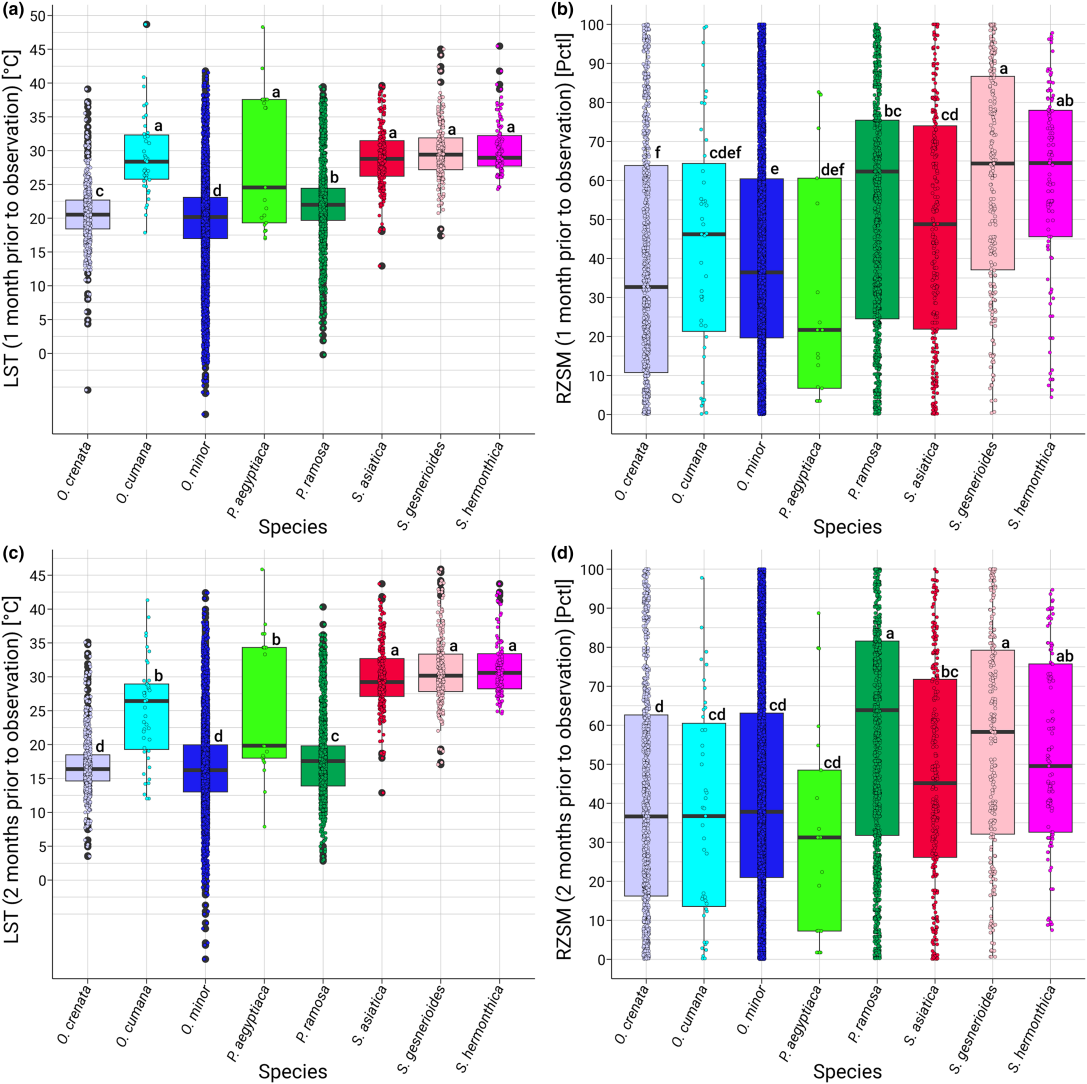
Temperature and moisture conditions across parasitic weed locations. (a, c) Land surface temperature and (b, d) root‐zone soil moisture conditions during 1 and 2 months prior to the plant's observations of different *Orobanche*, *Phelipanche*, and *Striga* species. Data are aggregated for the years 2006–2022. Boxplots visualize the distribution of observed elevations per year for different species between 2006 and 2022. Central lines represent median, box edges correspond to the first (Q1) and third quartiles (Q3), whiskers represent the range of elevation data within 1.5 times the interquartile range (IQR), with any data points beyond this range marked as outliers. Dots represent individual observations. Significant differences in elevation between years are indicated by letters.

To gain insights into the environmental conditions, including extreme conditions, that are critical during the preconditioning and germination phases of the species studied, we investigated land surface temperature (LST) and root‐zone soil moisture (RZSM) at the locations where the plants were observed. In our study of LST associated with various species of *Striga*, *Orobanche*, and *Phelipanche*, we analyzed data from 1 and 2 months prior to plant observations. The analysis revealed that in both months, all species endured a wide range of temperature conditions, including high and low extremes. Despite this broad temperature spectrum, there were notable differences in mean LST values among the species, with *S. gesnerioides*, *S. hermonthica*, and *S. asiatica* consistently exhibiting higher mean LSTs compared to *O. cumana*, *O. crenata*, and *O. minor*. This distinction was even more pronounced when considering the 75th percentile values, which further underscored the higher temperature preference of *Striga species*. In the genus *Phelipanche*, *P. aegyptiaca* had higher mean and 75th percentile temperature values compared to *P. ramosa*, also reflecting their different predominant locations (Table 1, Figure 2a,b).

**TABLE 1:** Land surface temperature (LST) and root‐zone soil moisture (RZSM) at locations where plants were observed.

	*Orobanche crenata*	*O. cumana*	*O. minor*	*Phelipanche aegyptiaca*	*P. ramosa*	*Striga asiatica*	*S. gesnerioides*	*S. hermonthica*
LST (1 month prior to observation) [°C]								
Mean	20.6	29.2	19.7	28.8	22.3	28.9	29.8	30.2
Min	−5.4	17.9	−9.0	17.0	−0.2	12.9	17.4	24.3
Max	39.1	48.7	41.8	48.3	39.5	39.6	45.0	45.5
Q3	22.7	31.9	23.1	37.6	24.4	31.5	31.9	32.2
Significance	c	a	d	a	b	a	a	a
LST (2 months prior to observation) [°C]								
Mean	16.8	24.9	16.6	26.0	17.9	29.9	31.0	31.4
Min	3.5	12.0	−11.7	7.9	2.8	12.9	17.1	24.6
Max	35.1	41.3	42.4	45.9	40.3	43.7	45.9	43.7
Q3	18.5	29.0	20.0	34.3	19.8	32.7	33.4	33.4
Significance	d	b	d	b	c	a	a	a
LST (1 month prior to observation) [Percentile]								
Mean	38.7	44.0	42.0	32.4	53.7	48.7	60.7	59.5
Min	0.1	0.1	0.0	3.5	0.2	0.1	0.4	4.4
Max	100.0	99.4	100.0	82.6	100.0	100.0	100.0	97.8
Q3	63.8	64.3	60.4	60.6	75.4	74.0	86.7	78.0
Significance	f	cdef	e	def	bc	cd	a	ab
LST (2 months prior to observation) [Percentile]								
Mean	40.6	37.2	43.1	31.0	56.6	46.9	55.2	52.6
Min	0.1	0.2	0.0	1.8	0.2	0.1	0.7	7.5
Max	100.0	97.8	100.0	88.7	100.0	100.0	100.0	94.7
Q3	62.6	60.5	63.1	48.5	81.6	71.8	79.2	75.7
Significance	d	cd	cd	cd	a	bc	a	ab

*Note*: Significant differences are indicated by letters.

In examining RZSM 1 and 2 months prior to the observation of various species of *Striga*, *Orobanche*, and *Phelipanche*, our analysis revealed that all species experienced a remarkably wide range of moisture conditions, spanning from dry levels (almost 0%) to full saturation (100%). Despite this broad range, we observed notable differences in mean moisture levels across species, which were even more pronounced when considering the 75th percentile values. The three *Striga* species consistently showed higher mean moisture levels and higher 75th percentile values compared to the three *Orobanche* species. Furthermore, *P. ramosa* exhibited higher mean and 75th percentile moisture values compared to *P. aegyptiaca*, indicating a greater tolerance or preference for more moist conditions by *P. ramosa*. In contrast, *P. aegyptiaca*, with lower mean and 75th percentile values, appears more adapted to drier conditions (Table 1, Figure 2c,d).

These results align with the ecological and geographical distributions of these species. *Striga* species are predominantly found in the warmer climates of the African Savannah, while *Orobanche* are more common in the varied, often cooler, Mediterranean climates.

### 3.2 *Striga* and *Orobanche* exhibit divergent temporal trends at high altitudes

**FIGURE 3:**
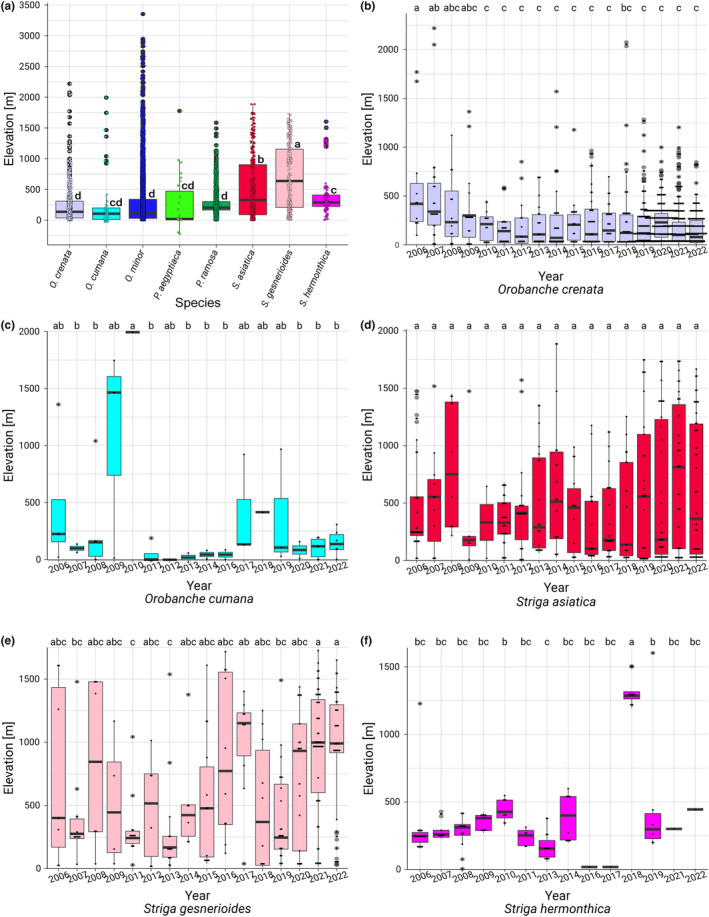
*Striga* and *Orobanche* exhibit divergent altitudinal trends. (a–f) Elevation values of plant observation locations for different *Orobanche*, *Phelipanche*, and *Striga* species. Boxplots visualize the distribution of observed elevations per year for different species between 2006 and 2022. Central lines represent median, box edges correspond to the first (Q1) and third quartiles (Q3), whiskers represent the range of elevation data within 1.5 times the interquartile range (IQR), with any data points beyond this range marked as outliers. Dots represent individual observations. Significant differences in elevation between years are indicated by letters.

We then correlated observations of *Striga*, *Orobanche*, and *Phelipanche* with altitude. All three *Striga* species were found at higher locations than *Orobanche* or *Phelipanche*, judged by the mean elevation values. However, *O. cumana* and *O. minor*, despite the lowest mean elevations, displayed the highest maximum elevations in the study. This reflects a remarkable capacity to adapt to a very wide range of elevations, extending from lowlands to significant heights. *Phelipanche aegyptiaca* and *P. ramosa* predominantly occupied lower elevation ranges, as suggested by their lower mean and quartile values, indicating a more defined ecological niche at lower altitudes (Table 2, Figure 3a). These results reflect the different habitats of *Striga* and *Orobanche* and between different species in the same group. For instance, the difference in altitudinal distribution between *S. hermonthica*, predominantly found in the flat Sahel region, and *S. asiatica*, which is more common in the highland‐rich areas of southern Africa and Asia, likely accounts for their observed variations in altitude preferences.

**TABLE 2:** Altitudinal locations of different *Striga*, *Orobanche*, and *Phelipanche* species.

Species	Altitude [m]				
	Mean	Min	Max	Q3	Significance
*Orobanche crenata*	221	−1	2220	309	d
*Orobanche cumana*	284	−28	1994	199	cd
*Orobanche minor*	240	−4	3353	339	d
*Phelipanche aegyptiaca*	260	−223	1777	471	cd
*Phelipanche ramosa*	254	−6	1585	303	d
*Striga asiatica*	523	−2	1886	904	b
*Striga gesnerioides*	698	8	1726	1154	a
*Striga hermonthica*	404	4	1603	410	c

*Note*: Significant differences are indicated by letters.

We then investigated the temporal trends in high‐altitude occurrences (elevations greater than 1000 m) for different species from 2006 to 2022 using logistic regression analysis. Over this period, *O. crenata* demonstrated a significant decrease in high‐altitude occurrences over time (Table 3, Figure 3b), while the negative trend for *O. cumana* was not statistically significant (Figure 3c). No statistically significant trends in high‐altitude occurrences were detected for *O. minor*, *P. ramosa*, and *P. aegyptiaca* respectively. In contrast, *S. asiatica* (Figure 3d) and *S. gesnerioides* (Figure 3e) showed a significant increase in the odds of high‐altitude occurrence. *Striga hermonthica* demonstrated an even stronger positive trend, which, however, should be treated with caution due to the very low number of data points above an elevation of 1000 m (Figure 3f).

**TABLE 3:** *Striga* and *Orobanche* exhibit divergent temporal trends at high altitudes.

Species	Log‐odds estimate	Standard error of estimate	*Z*‐score	*p*‐value
*Orobanche crenata*	−0.203149271	0.045274335	−4.487073582	.00001
*Orobanche cumana*	−0.324848496	0.168094447	−1.932535559	.05329
*Orobanche minor*	−0.021683582	0.012151146	−1.78448862	.07434
*Phelipanche aegyptiaca*	18.44050007	7073.356643	0.002607037	.99792
*Phelipanche ramosa*	0.035931065	0.087233081	0.411897236	.68041
*Striga asiatica*	0.097660201	0.02844763	3.43298194	.0006
*Striga gesnerioides*	0.081935332	0.030634815	2.674582256	.00748
*Striga hermonthica*	0.439099338	0.104220513	4.213175767	.00003

*Note*: Results are shown of the logistic regression models fitted for each species for the period 2006–2022 for occurrence at elevations greater than 1000 m. Log‐odds estimate indicates the expected change in the log‐odds for each additional year, standard error of estimate refers to the precision of this estimate, *Z*‐score shows the significance of the estimate by indicating its distance from zero in standard error units, and *p*‐value reflects statistical significance.

In summary, *Striga* asiatica and Striga *gesnerioides* displayed a significant temporal trend towards higher elevations, whereas *Orobanche crenata* demonstrated the opposite.

### 3.3 Interplay of temperature, moisture, and elevation in Orobanchaceae habitats

**FIGURE 4:**
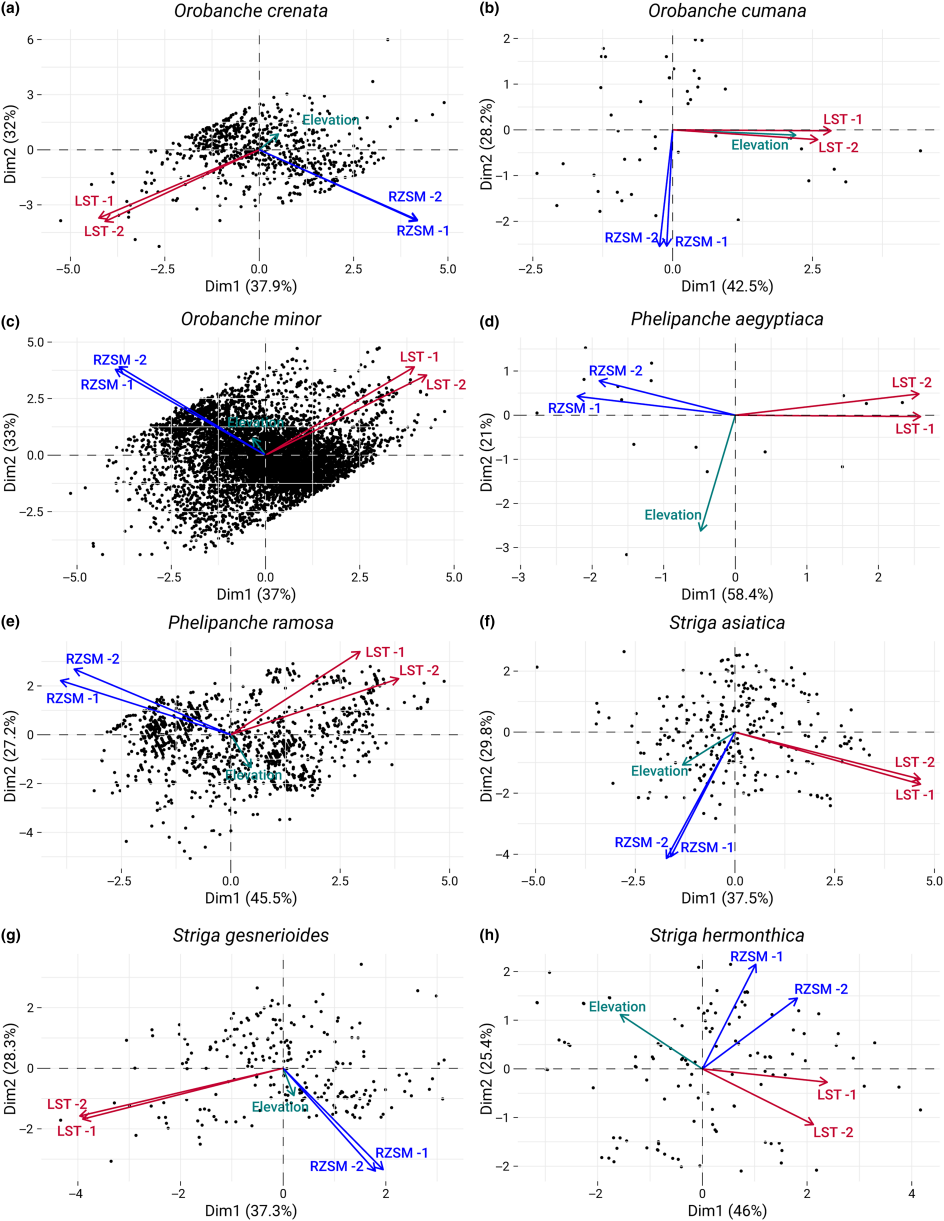
Principal component analysis (PCA) biplots illustrate the relationship between individual observations and the environmental variables elevation, land surface temperature (LST), and root‐zone soil moisture (RZSM) for different Orobanchaceae species (a–h). Points represent individual observations. Arrows demonstrate the influence of environmental variables, with direction and length indicating their relationship to the principal components.

We then conducted a principal component analysis (PCA) on the eight Orobanchaceae species in this study to understand the correlations between the environmental variables LST, RZSM, and elevation and their potential influence on the ecological adaptations and habitat preferences of these species. We found a slightly negative correlation of LST and RZSM in *O. crenata* and *O. minor*, but no correlation in *O. cumana*. Instead, in *O. cumana*, we found a strong correlation of LST and elevation (Figure 4a–c). Of the three genera, the two *Phelipanche* species showed the strongest negative correlation between root‐zone soil moisture and land surface temperature (Figure 4d,e). For the three *Striga* species, LST and RZSM were largely uncorrelated. However, we noticed a clear correlation of RZSM and elevation for *S. asiatica* and *S. gesnerioides*, and a clear negative correlation of LST and elevation for *S. hermonthica* (Figure 4f–h).

### 3.4 Host availability drives high‐altitude occurrences of *O. crenata* but not of *S. asiatica*

We examined how closely the habitats of *Striga asiatica* and *Orobanche crenata* are shaped by the presence of their host plants at their respective locations. Due to substantial differences in aggregation methods and crop categorizations between Earthstat and GAEZ+ datasets, direct quantitative comparisons of harvested areas and yields were challenging. Therefore, our analysis employed a conservative approach: using Earthstat 2000 data, we verified the presence of potential host plant species at each parasitic plant observation site. We cross‐referenced these findings with the 2015 GAEZ+ data to check for the persistence or emergence of these host plants. We compared the geolocations of *O. crenata* with crop data for beans, peas, and lentils from the year 2000, and the geolocations of *S. asiatica* with the crop data for maize, sorghum, millet, and rice. We found that at least one host crop plant was present in 84% of all *O. crenata* locations and in 100% of its locations at altitudes higher than 1000 m. When we compared these numbers to crop data from the year 2015, we found that while overall host presence at *O. crenata* locations had increased to 96%, it had decreased to 35% at locations at an altitude of 1000 m or higher. We did the same analysis for *S. asiatica* occurrences and found relatively stable values with 84% host presence at all locations in the year 2000 and 83% in the year 2015. At locations at altitudes greater than 1000 m, this number changed from 93% in the year 2000 to 88% in the year 2015 (Table 4).

**TABLE 4:** Presence of host plants at locations where parasitic plants were observed.

	2000	2015
*Orobanche crenata*		
All	92%	96%
Elevation >1000 m	100%	35%
*Striga asiatica*		
All	84%	83%
Elevation >1000 m	93%	88%

These findings indicate that the downwards trend for high altitude observations for *O. crenata* is likely driven by the decrease of its hosts presence at these locations. In contrast, host plants for *S. asiatica* have been present prior to its occurrence at high‐altitude locations. This suggests that while host availability provides the necessary conditions for the presence of *S. asiatica*, it does not appear to be the primary driver of the observed upwards trend at high altitudes.

### 3.5 *Striga asiatica* observations are closely associated with heat‐moisture coupling

**FIGURE 5:**
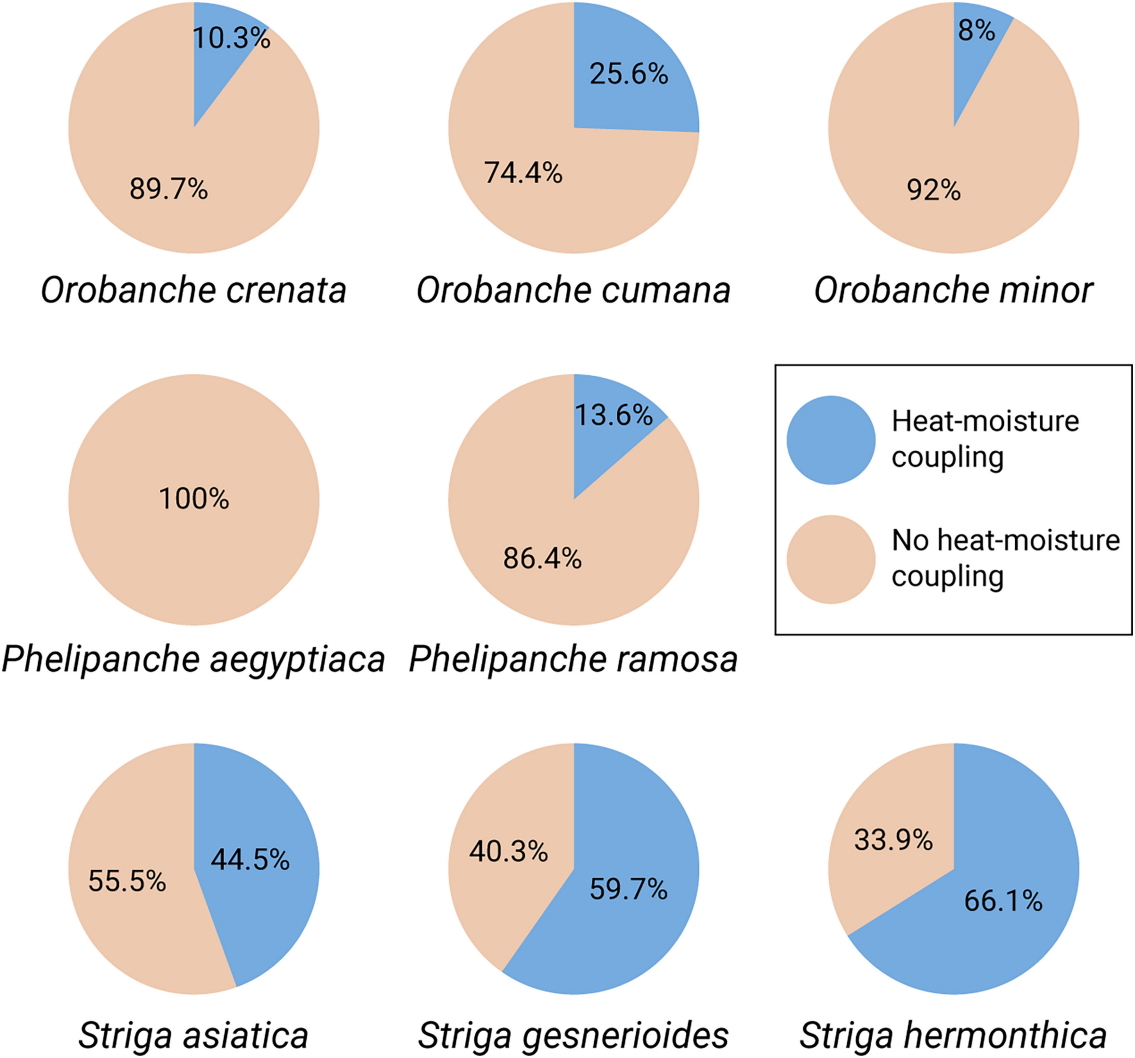
Striga observations are closely associated with preceding heat‐moisture couplings. Pie charts show the proportion of observations for each species where heat‐moisture coupling events occurred (land surface temperature ≥25°C, root zone soil moisture percentile ≥70) at any point (month of the plant observation, or 1 or 2 months prior to observation).

*Striga*'s optimal seed preconditioning response to higher temperature and moisture levels suggests that an interplay of these two factors is crucial and that the absence or insufficiency of one might inhibit germination entirely. We therefore investigated the co‐occurrence of high LST and RZSM to capture a potentially optimal environmental window that could favor germination and subsequent emergence of these parasitic plants. We assessed the association between the occurrence of different *Striga*, *Orobanche*, and *Phelipanche* species and preceding heat‐moisture coupling events, defined as conditions where the land surface temperature (LST) was equal to or exceeded 25°C and the root zone soil moisture (RZSM) was in the 70th percentile or above in the month of or in the 2 months prior to a plant's observation. In our dataset, 66.1% of *S. hermonthica*, 59.7% of *S. gesnerioides*, and 44.5% of *S. asiatica* observations followed a heat‐moisture coupling event. Comparatively, *Orobanche* species displayed less association, with 25.6% of *O. cumana*, 10.3% of *O. crenata*, and 8.0% of *O. minor* observations recorded after a heat‐moisture coupling event. Among the *Phelipanche* species, we found no instances where *P. aegyptiaca* occurrences were preceded by a heat‐moisture coupling event, while 13.6% of *P. ramosa* observations followed such an event (Figure 5).

While the distinct climatic ranges of these species are known, our results quantify how these differences manifest in the context of heat‐moisture couplings.

### 3.6 Heat‐moisture coupling events rise significantly at high‐altitude *Striga asiatica* sites

**FIGURE 6:**
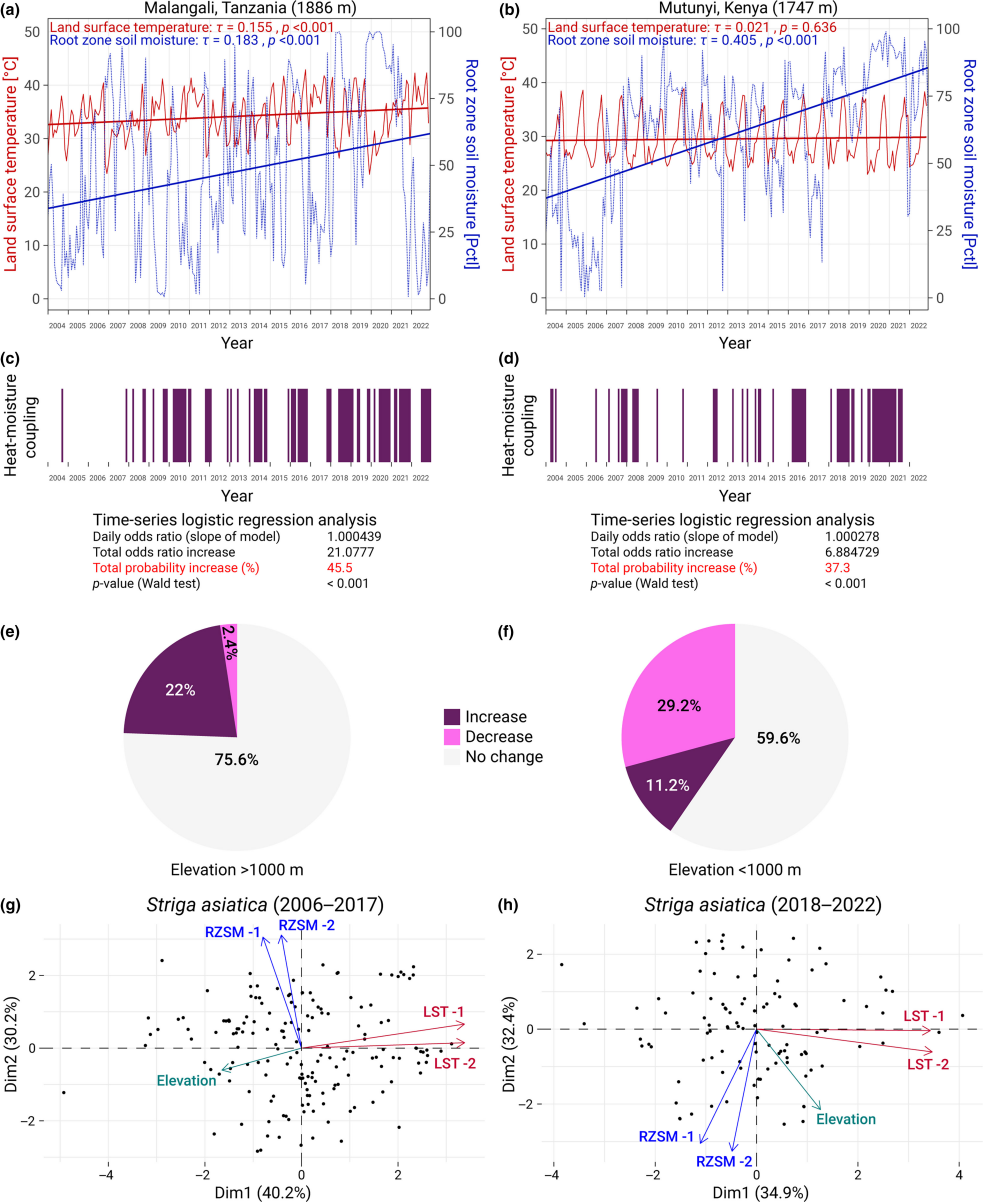
High‐altitude locations of *Orobanche crenata* experience decreased soil moisture, and those of *Striga asiatica* experience a rise in heat‐moisture couplings. (a,b) Time‐series plots illustrating temporal variations of land surface temperature and root‐zone soil moisture at given locations. Superimposed linear trendlines denote overall trend of data over time, while statistics from the Mann‐Kendall test, tau (*τ*) and *p*‐value, indicate the trend's strength, direction, and statistical significance respectively. (c,d) Heatmaps indicating the temporal pattern of heat‐moisture coupling events for the locations in (c) and (d), where a purple column represents the occurrence of an event. Accompanying these visualizations are results from logistic regression models that assess the temporal trend in these events' occurrences. The calculated statistics include the model's slope (which indicates the change in the log‐odds of a heat‐moisture coupling event for each unit increase in time), the total increase in these odds over the study period, the total increase in the probability of such an event over time, and the corresponding *p*‐values from the Wald test, which determine the statistical significance of these changes over time. (e,f) Percentages of increases and decreases of heat‐moisture coupling trends for all locations of *Striga asiatica* observations, showing a separate analysis for locations at elevations greater and smaller than 1000 m. (g,h) PCA biplots illustrate the relationship between individual observations and the environmental variables elevation, LST, and RZSM for *Striga asiatica* in two different time periods. Points represent individual observations. Arrows demonstrate the influence of environmental variables, with direction and length indicating their relationship to the principal components.

Upon further exploration of the varying responses to heat‐moisture coupling events exhibited by *S. asiatica*, we evaluated monthly LST and RZSM trends at specific high‐altitude locations. These areas were selected based on the observed preferences of *S. asiatica*, which showed an increased likelihood of high‐altitude occurrences. We conducted Mann‐Kendall tests for monotonic trends for two high‐altitude sites of *S. asiatica*: At location 1 in Malangali, Tanzania (1886 m altitude), we found a significant upward trend in both LST and RZSM.(Figure 6a). At location 2 in Mutunyi, Kenya (1747 m altitude) the LST trend was negligible, however the RZSM demonstrated a significantly positive trend (Figure 6b).

We further investigated the trend in the frequency of these events at these locations by performing a logistic regression analysis on the time‐series data. At location 1 in Malangali, Tanzania, the logistic regression model demonstrated a statistically significant trend, with a total increase in the odds of a heat‐moisture coupling event of approximately 21.1 times, which equals an aggregate increase in the likelihood by 45.5% (Figure 6c). Similar trends were observed at location 2 in Mutunyi, Kenya, where the estimated daily odds increase was approximately 6.9 times, or an overall probability increase of 37.3% (Figure 6d).

We then examined all reported locations of *S. asiatica* for a potential increase or decrease of heat‐moisture coupling events. For an elevation of greater than 1000 m, we found that 22% of locations are undergoing an increase and 2.4% a decrease (Figure 6e). These numbers were substantially different for locations at an elevation under 1000 m, with 11.2% of locations undergoing an increase of heat‐moisture couplings and 29.2% a decreasing trend (Figure 6f).

Finally, we conducted a principal component analysis examining if there had been a change in correlation of elevation with either LST or RZSM in *S. asiatica*. When we analyzed *S. asiatica* occurrences between 2006 and 2017, we saw a strong negative correlation between elevation and LST and no correlation between elevation and RZSM (Figure 6g). We then examined data points between 2018 and 2022 (for which we had already noticed higher altitude occurrences, see Figure 3d). Again, we found no correlation bet LST and RZSM; however, we now found elevation having a correlation to both LST and RZSM (Figure 6h).

These results demonstrate that there has been a marked increase in the frequency of heat‐moisture coupling events at high‐altitude locations of *S. asiatica*, suggesting possible interactions between climate factors and the geographical distribution of this species. These data are consistent with our earlier results showing an increased occurrence of *S. asiatica* at high altitudes.

## 4 DISCUSSION

In this study, we sought to gain insight into environmental preferences of *Orobanche*, *Phelipanche*, and *Striga*, and the effect these preferences have on their geographic distribution (see Figure 1a for a global overview).

In our study, we utilized GBIF data for Orobanchaceae occurrences and correlated them with various environmental variables using different satellite‐based datasets, each with unique strengths and weaknesses. While older records in the GBIF database sometimes present challenges due to inaccurate or missing geolocation information, we did not find this to be an issue for our data from 2001 onwards. The Shuttle Radar Topography Mission (SRTM) offers high‐resolution elevation data, though its accuracy may be reduced in densely vegetated or urban areas, and it does not reflect changes post‐2000. MODIS land surface temperature (LST) data provides comprehensive temperature information but can be affected by atmospheric conditions and cloud cover, with a resolution that may not capture minute local variations. Root‐zone soil moisture (RZSM) data, while crucial for understanding soil conditions, relies on model calibrations and satellite observations, which may not always represent small‐scale soil moisture variations accurately. Therefore, while these datasets are generally reliable, their inherent limitations necessitate careful interpretation.

When we examined environmental parameters such as land surface temperature and root zone soil moisture, we observed significant differences in the ecological habitats of *Striga* and *Orobanche* species. *Orobanche crenata* and *O. minor* were associated with cooler and comparatively drier environments, corroborating their preference for moderate temperatures during the preconditioning stage (Matusova et al., 2004). These species demonstrated moderate LST values, consistent with their primarily temperate habitat, and moderate RZSMs, suggesting their ability to thrive in drier soil conditions. On the other hand, *S. hermonthica* and *S. gesnerioides* were found in warmer and moister environments, aligning with their optimal preconditioning response to higher temperatures and moisture levels (Aflakpui et al., 1998; Babiker et al., 2001; Igbinnosa & Okonkwo, 1992). While *S. hermonthica* seeds prefer warm and moist environments for preconditioning, it should be mentioned that there seems to be a limit to the beneficial effects of rainfall or soil moisture. A recent study demonstrated that a peak *S. hermonthica* seedbank was associated with annual rainfall levels around 549 mm per annuum, and a decline in the seedbank was observed when yearly precipitation surpassed 600 mm (Aliyu et al., 2023). This result could suggest that excessive soil moisture levels may prove unfavorable for the species' propagation. Another critical aspect to consider is the potential dilution effect on germination stimulants, particularly strigolactones, in the rhizosphere. *Striga* seeds rely on these stimulants, exuded by host plant roots, to trigger germination. Excessive soil moisture would dilute the concentration of these stimulants, reducing their effectiveness. Additionally, higher water levels can alter soil pH, diluting a plant's ability to acidify the rhizosphere and thereby affecting the stability of strigolactones, which are known to require a certain level of acidity to remain stable (Zwanenburg & Pospisil, 2013). This pH dilution could further decrease the availability of effective stimulants.

When we investigated the species' altitudinal distribution, we not only observed that *S. asiatica* and *S. gesnerioides* were found to thrive at considerably higher altitudes than the other species but that a gradual propensity for higher altitude environments was apparent in *S. asiatica* and *S. gesnerioides*. In contrast, *Orobanche* species, predominantly found in the Mediterranean basin, did not exhibit a preference for higher altitudes. Instead, they were more commonly found in lower elevation regions characterized by a generally mild climate, which is distinct from the typically hotter savannah conditions, the primary habitat for most *Striga* (see also Figure 1b). Notably, *O. crenata* showed a significant decrease in high‐altitude occurrences over time, likely driven by the disappearance of available host plants. The results from the principal component analysis underscore these habitats. For example, the strong correlation of LST and elevation in *O. cumana* reflects their high‐elevation occurrences in the higher temperature regions central Spain and the Middle East, compared to their lower altitude and colder habitats in Ukraine and Russia. The opposite trend for *S. hermonthica* (strong negative correlation between LST and elevation) can be explained with the respective habitats as well. The higher altitude locations in east and southern Africa are characterized by milder temperatures than the hot temperatures in the lowlands of the Sahel zone.

Analyzing the association between different *Striga*, *Orobanche*, and *Phelipanche* species and preceding heat‐moisture coupling events, we noticed significant interspecies differences. We defined heat‐moisture coupling events as circumstances where land surface temperature (LST) hits or exceeds 25°C, and the root‐zone soil moisture (RZSM) percentile reaches 70 or higher, in the plant observation month or the 2 months prior. *Striga* species, such as *S. hermonthica* (66.1%), *S. gesnerioides* (59.7%), and *S. asiatica* (44.5%), exhibited a marked correlation with these events. This aligns with prior knowledge about *Striga* species favoring higher temperature and moisture levels for optimal preconditioning, thus aiding in seed germination (Aflakpui et al., 1998; Babiker et al., 2001). In contrast, *Orobanche* species, including *O. cumana* (25.6%), *O. crenata* (10.3%), and *O. minor* (8.0%), showed a weaker association with these climatic events, reflecting their preference for moderate temperature conditions. Among the *Phelipanche* species, no instances of *P. aegyptiaca* occurrences followed a heat‐moisture coupling event, while only 13.6% of *P. ramosa* observations were noted after such an event. This is in agreement with previous observations that *P. aegyptiaca* and *O. cumana* do not require a preconditioning phase for seed germination (Plakhine et al., 2009). These observations also suggest that specific climatic conditions could influence the distribution patterns of these parasitic weeds. *Striga* species, in particular, could experience potential geographical shifts under climate change if the frequency of heat‐moisture coupling events increases.

For this reason, we examined climatic trends at specific high‐altitude habitats of *S. asiatica* and *O. crenata*. While the downward trend of *O. crenata* occurrences at high altitudes coincided with the receding availability of host plants, this was not the case for *S. asiatica*. Here, we did not find any changes in host availability, instead, high‐altitude areas hosting *Striga asiatica* experienced an increased frequency of heat‐moisture coupling events, making the occurrence of a heat‐moisture‐coupling significantly more likely at the present time than 19 years ago. These observed trends might provide an explanation for the observed expansion of *S. asiatica* into higher‐altitude habitats. The contrasting trends at high‐altitude locations further emphasize the species‐specific responses of *S. asiatica* and *O. crenata* to changing environmental conditions, suggesting that the potential geographical shifts predicted by several studies are already underway.

In this study, one challenge was comparing the 2000 Earthstat and 2015 GAEZ+ datasets, particularly with respect to harvested areas. These datasets not only differ in their categorization of crops but also in their methodologies for calculating harvested areas. Such variations precluded us from drawing precise conclusions about changes in cultivation density or the specific extents of crop areas over time. Consequently, we focused on the presence of host plants as a more reliable and consistent indicator for our analysis of the distribution of parasitic plants. While the expansion of cultivation at higher altitudes likely influences the distribution of these parasitic species, our datasets did not provide the necessary detail to explore this aspect. This limitation highlights the need for more uniform and detailed agricultural data in future research. Additionally, while our study focused on specific environmental parameters, we acknowledge the potential influence of other factors such as topography, wind patterns, and soil properties on species distribution. Topographical features, beyond elevation, such as slope and aspect, can create microclimatic variations that may affect habitat suitability. Wind patterns are crucial for species with airborne seeds, influencing both dispersal mechanisms and local climate conditions. Soil properties, including changes in nutrient content due to varying fertilizer use, can also impact the emergence of parasitic plants. While our dataset and the scope of our study did not permit an extensive analysis of these variables, their potential roles highlight important areas for future research.

Our study underscores the significance of climate factors in influencing the distribution of parasitic plants. As climate change continues to modify these factors, we can anticipate shifts in the geographical distribution of these plants, highlighting the need for continuous monitoring and adaptive strategies to mitigate their impact on global agriculture.

## AUTHOR CONTRIBUTIONS

**Marco Bürger:** Conceptualization (equal); data curation (equal); formal analysis (equal). **Joanne Chory:** Conceptualization (equal).

## CONFLICT OF INTEREST STATEMENT

The authors have no conflict of interests to disclose.

## Data Availability

Data on the occurrences of the *Orobanche*, *Phelipanche*, and *Striga* species were obtained from the Global Biodiversity Information Facility (GBIF). The data were accessed and downloaded on the 2nd of May, 2023 using the following datasets: For *Orobanche* species: GBIF Occurrence Download https://doi.org/10.15468/dl.hdjf3h. For *Phelipanche* species: GBIF Occurrence Download https://doi.org/10.15468/dl.sn54fx. For *Striga* species: GBIF Occurrence Download https://doi.org/10.15468/dl.2hdkfj. The R code used for data analysis and generating results can be accessed on GitHub (https://github.com/plantxray/plantxray.github.io/tree/main/ECE3_11332). Any additional data or materials related to this study are available from the corresponding author upon reasonable request.
